# Reciprocal Substitution Between Methamphetamine and Heroin in Terms of Reinforcement Effects in Rats

**DOI:** 10.3389/fpsyt.2020.00750

**Published:** 2020-07-31

**Authors:** Di-sen Mei, Yu-jia Cai, Fang-min Wang, Bao-miao Ma, Hui-fen Liu, Wen-hua Zhou, Jiang-ping Xu

**Affiliations:** ^1^ Neuropharmacology and Drug Discovery Group, School of Pharmaceutical Sciences, Southern Medical University, Guangzhou, China; ^2^ Zhejiang Provincial Key Lab of Addiction, Ningbo University School of Medicine, Ningbo, China; ^3^ Laboratory of Behavioral Neuroscience, Ningbo Addiction Research and Treatment Center, Ningbo University School of Medicine, Ningbo, China

**Keywords:** amphetamine, opiate, drug abuse, conditioning reward, addiction

## Abstract

Heroin and methamphetamine are both popular illicit drugs in China. Previous clinical data showed that habitual users of either heroin or methamphetamine abuse the other drug for substitution in case of unavailability of their preferred drug. The present study aimed to observe whether heroin can substitute the methamphetamine reinforcement effect in rats, and vice versa. Rats were trained to self-administer heroin or methamphetamine (both 50 μg/kg/infusion) under an FR1 reinforcing schedule for 10 days. After having extracted the dose–effect curve of the two drugs, we administered methamphetamine at different doses (12.5–200 μg/kg/infusion) to replace heroin during the period of self-administration, and vice versa. The heroin dose–effect curve showed an inverted U-shaped trend, and the total intake dose of heroin significantly increased when the training dose increased from 50 to 100 or 200 μg/kg/infusion. Following replacement with methamphetamine, the total dose–effect curve shifted leftwards and upwards. By contrast, although the dose–effect curve of methamphetamine also showed an inverted U-shaped trend, the total dose of methamphetamine significantly decreased when the training dose decreased from 50 to 25 μg/kg/infusion; conversely, when the methamphetamine training dose increased, the total dose did not change significantly. The total dose–effect curve shifted rightwards after heroin was substituted with methamphetamine. Although heroin and methamphetamine had their own independent reward effects, low doses of methamphetamine can replace the heroin reward effect, while high doses of heroin can replace the methamphetamine reward effect. These results demonstrated that heroin and methamphetamine can substitute each other in terms of reinforcement effects in rats.

## Introduction

Drug abuse remains a significant public health concern in China and worldwide. The 2016 Annual National Drug Report of China counted about 2.95 million drug abusers registered in China; moreover, real drug users were estimated to be more than 14 million (1). Before 2014, heroin was the most common illicit drug in China, followed by methamphetamine (MA), either in the form of crystals or pills; henceforth, the number of MA users increased significantly coinciding with the reduction of heroin users (1), suggesting dual drug use epidemics in China. In addition, issues related to the combined utilization of MA and heroin were also identified (2). Further, a 2014 survey conducted in five major Methadone Maintenance Treatment (MMT) clinics in the Yunnan province showed that the urine of about 9.2% of participants was positive for both morphine and MA (3). Previous clinical data showed that among current heroin users, half of them used amphetamine during the preceding 6 months while, in the case of amphetamine users, two-thirds of them used heroin in that period; besides, although transitions from amphetamines to heroin are more common than those in the other way round, they are relatively common in both directions (4). A 2015 survey from the National HIV Behavioral Surveillance (NHBS) system in Denver, Colorado, showed that half of the participants injected both MA and heroin during the past 12 months (5). Recently, the last-month use of MA among treatment-seeking opioid users significantly increased from 18.8% in 2011 to 34.2% in 2017 (6). Since the global emergence of combining MA with heroin may have serious public-health implications, understanding the mechanisms underlying co-morbidity of MA and heroin use disorders is crucial.

Heroin is a µ-opioid receptor agonist that mimics the effects of endogenous opioids, resulting in euphoria, analgesia, and sedation (7). On the other hand, MA — a psychostimulant — principally affects the monoamine neurotransmitter system, resulting in alertness feelings, increased energy, and euphoria (8). Heroin and MA greatly differ in many aspects, including their pharmacological features, behavioral or neurobiological mechanisms, and clinical effects (9, 10); however, heroin and MA share the ability to increase dopamine levels in the nucleus accumbens (NAc), one of the terminal regions of the mesocorticolimbic dopamine projecting system; the activation of this system and the subsequent dopamine release play a key role in the rewarding effects of the drugs (11). Interestingly, heroin is an attractive option for MA users due to its high purity and relatively low price (12). Similarly, opioids users prefer MA when it is easily accessible or cheap (6). Nonetheless, the reasons for the combined utilization of MA and heroin are still unclear.

The present study investigates whether heroin and MA can substitute each other in terms of reinforcement effect in rats. First, we observed whether MA could replace heroin reinforcement effects in heroin intravenous self-administered rats. Second, we evaluated whether heroin could substitute MA reinforcement effects in MA self-administered rats.

## Materials and Methods

### Animals and Drugs

Male Sprague–Dawley rats initially weighing 260 to 300 g(Zhejiang Experimental Animal Center, Hangzhou, China) were individually housed in a temperature-controlled and ventilated colony room, with food and water available *ad libitum.* To simulate the rodents’ natural state, all experiments were conducted during the dark phase of the light–dark cycle (lights on from 7:30 am to 6:30 pm). We performed the experiments following the Guide for the Care and Use of Laboratory Animals by the National Institutes of Health (8th edition).

We used heroin (diacetylmorphine HCl) and D-methamphetamine — obtained from the National Institute of Forensic Science (Beijing, China) — dissolved in sterile 0.9% physiological saline.

### Surgery

The rats were anesthetized with sodium pentobarbital (50 mg/kg, i.p.) and implanted with chronically indwelling jugular catheters as previously described (13, 14). The catheter was flushed daily with 0.3 ml saline containing heparin (5 units) and penicillin B (20,000 units) to prevent bacterial infection and maintain catheter patency. Following the surgery, the rats were allowed 7 d to recover.

### Intravenous Self-Administration Training

The rats were trained to self-administer heroin or MA in operant chambers following an FR1 schedule for 4 h/d during 10 consecutive days. Every session started with a blue light inside the “active” nose-poke hole. The rat received a single infusion following a nose-poke inside the “active” hole based on an FR1 schedule. Each infusion was paired with 5 s of illumination at room light, as well as with the noise from the infusion pump, as a discrete conditioned stimulus (CS). Further, we set a timeout period of 20 s during which the response produced no programmed consequences but was still recorded. Illumination of the blue light in the “active” nose-poke signaled the end of the timeout period. Nose-poking in the “inactive” hole produced no programmed consequences.

#### Special Experiments

##### Experiment 1: MA Substitution of the Heroin Reinforcement Effects

*Heroin self-administration*: All rats (n=19) were trained to self-administer heroin for 10 days (4 h/d; 100 μg/kg/infusion for the first 3 days, 50 μg/kg/infusion for last 7 days) based on an FR1 reinforcement schedule.

*Dose–effect response in heroin self-administration*: On the 11th day, the rats were randomly divided into three groups (n = 6–7/group). Each group self-administered heroin for 4 h with doses of either 5, 12.5, or 25 μg/kg/infusion instead of the original dose of heroin (50 μg/kg/infusion). On the 12th day, all rats recovered with the original dose of heroin. On the 13th day, the rats were randomly divided into two groups (n = 9–10/group) and each group was given either 100 or 200 μg/kg of heroin for 4 h.

*MA substituted for heroin reinforcement:* Following another recovery day, on the 15th day, the rats were randomly divided into two groups (n = 9–10/group) with the order mixed and counterbalanced. Each group self-administered MA doses of either 12.5 or 50 µg/kg/infusion for 4 h. Then, MA doses of 25 or 100 μg/kg/infusion were given for 4 h on alternate days, using the original heroin dose for recovery on interval days. Then in one groups (n=9) MA dose of 200 μg/kg/infusion were given for 4 h on alternate days.

##### Experiment 2: Heroin Substitution of the MA Reinforcement Effects

*MA self-administration:* All rats (n=16) were trained to self-administer MA for 10 days (4 h/d; 50 μg/kg/infusion) as described above.

*Dose–effect response in MA self-administration:* On the 11th day, the rats were randomly divided into two groups (n=8/group). Each group self-administered MA doses of either 25 or 100 μg/kg/infusion instead of the original dose of MA (50 μg/kg/infusion) for 4 h. On the 12th day, all rats recovered with the original dose of MA (50 μg/kg/infusion). On the 13th day, the rats were randomly divided into two groups (n = 7–8/group) and each group was given either 50 or 200 μg/kg of heroin for 4 h. One rat was abandoned due to an unexpected physical condition.

*Heroin substituted for MA reinforcement*: After 1 day for recovery, the rats were randomly divided into two groups (n = 5–6/group) with the order mixed and counterbalanced. Each group used a dose of either 12.5, 25 μg/kg/infusion heroin to replace the original dose of MA (50 μg/kg/infusion) for 4 h; then, the heroin doses changed to either 50 or 200 μg/kg/infusion on alternate days, using the original heroin dose (50 μg/kg/infusion) on recovery interval days.

### Data Statistics

Data are expressed as mean ± SEM. All statistic tests were performed using SPSS (IBM, Armonk, New York, United States). The mean amount of “active” and “inactive” pokes, infusions, and total dose during self-administration were analyzed *via* One-way ANOVAs with Bonferroni *post hoc* testing when appropriate. And the t test was used to compare between the two groups. A P-value< 0.05 was considered statistically significant.

## Results

### The Dose–Effect Curve of Heroin Self-Administration

**Figure 1:**
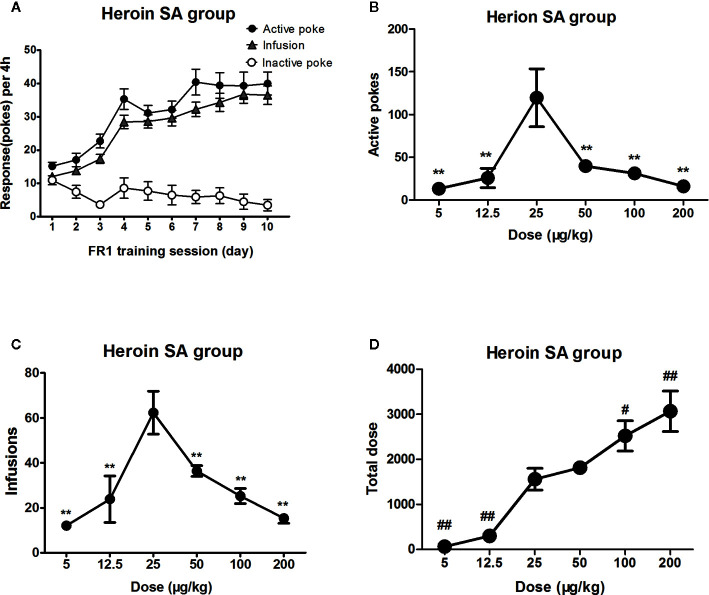
Dose–effect response in heroin self-administration. Rats (n = 19) were successfully trained to self-administer heroin 4 h/d under an FR1 reinforcing schedule within 10 days **(A)**. When the heroin trained rats were tested in different dose (5 μg/kg/infusion–200 μg/kg/infusion) of heroin to self-administer 4 h in the following days (n=6-10/group). The active pokes and the infusions were changed and showed an inverted U trend, whose peak likely being 25 μg/kg/infusion **(B, C)**. Rats took more heroin when doses increased to wither 100 or 200 μg/kg/infusion and less heroin intake when doses highly decreased to either 5 or 12.5 μg/kg/infusion, but maintained the total dose unchanged following the dose decreased to lower at 25 μg/kg/infusion **(D)**. **P < 0.01, compared to 25 μg/kg/infusion; ^#^ *P* < 0.05, ^##^ *P* < 0.01, compared to 50 μg/kg/infusion. Error bars = SEM.

Rats were successfully trained to self-administer heroin under an FR1 reinforcing schedule (**Figure 1A**). When in dose–effect response in heroin self-administration session, One-way ANOVA revealed a significant difference in the active pokes (F_5, 41_ = 7.6, *P*<0.01), infusions (F_5,41_ = 10.4, *P*<0.01), and total dose of heroin intake (F_5,41_ = 18.1, *P*<0.01). The *post hoc* comparisons indicated that at the number active pokes and infusions at the dose of 25 μg/kg/infusion was significantly increased compared to other doses. The dose–effect curve of heroin showed an inverted U-shaped trend, whose peak likely being 25 μg/kg/infusion. Nevertheless, the active pokes and the infusions of high dose of heroin changed not so significantly (**Figures 1B, C** **)**. Besides, the total dose of heroin intake showed a linear trend, being higher at high doses (i.e., 100 or 200 μg/kg/infusion) (**Figure 1D**).

### The Dose–Effect Curve of MA Substitution for Heroin Reinforcement

**Figure 2:**
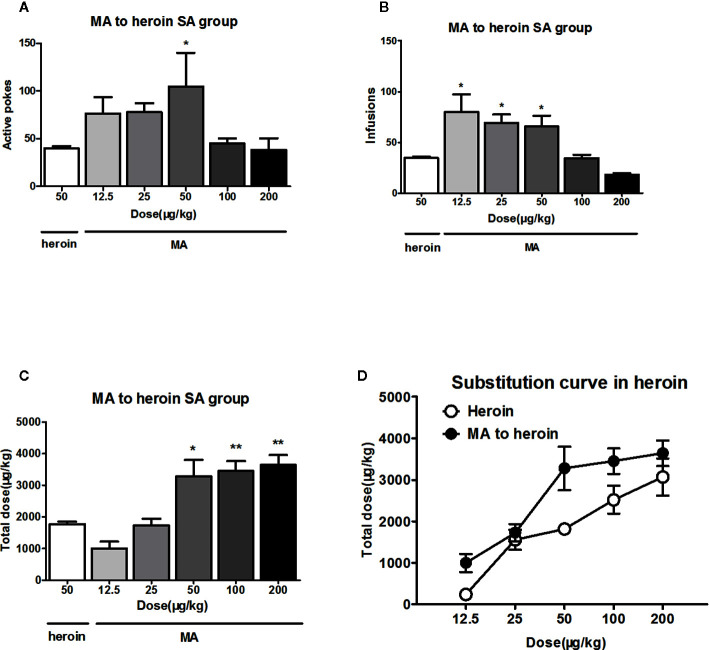
MA substituted for heroin reinforcement. As substitution with different dose of MA to self-administer 4 h in heroin trained rats (n=9-10/group), only dose of 50 μg/kg/infusion of MA significantly increased the active pokes and the lower doses of 12.5, 25, 50 μg/kg/infusion of MA increased the infusions **(A, B)**. The total dose significantly increased above the training MA dose of 50 μg/kg/infusion, and 25 μg/kg/infusion could equally instead of 50 μg/kg/infusion heroin self-administration at total dose **(C)**.The total dose–effect curve of MA replacement showed shifted leftward and upward compared to the total dose–effect curve of heroin **(D)**. *P < 0.05, **P < 0.01, compared to 50 μg/kg/infusion heroin. Error bars = SEM.

When the rats were allowed to self-administer several MA doses instead of heroin, they exhibited significant changes in terms of active pokes (F_5, 47 _= 2.7, *P*<0.05) and infusions (F_5, 47_ = 7.1, *P*<0.01). However, *post hoc* comparisons indicated that MA significantly increased the active pokes and the infusions only at low doses (**Figures 2A, B** **)**.

Moreover, multiple comparisons revealed that, when replaced with MA, the total intake infusions only significantly increased above the training MA dose of 50 μg/kg/infusion, and 25 μg/kg/infusion could equally instead of 50 μg/kg/infusion heroin self-administration at total dose (**Figure 2C**). The total dose curve of MA substitution shifted leftwards and upwards compared to the total dose–effect curve of heroin (**Figure 2D**).

### The Dose–Effect Curve of MA Self-Administration

**Figure 3:**
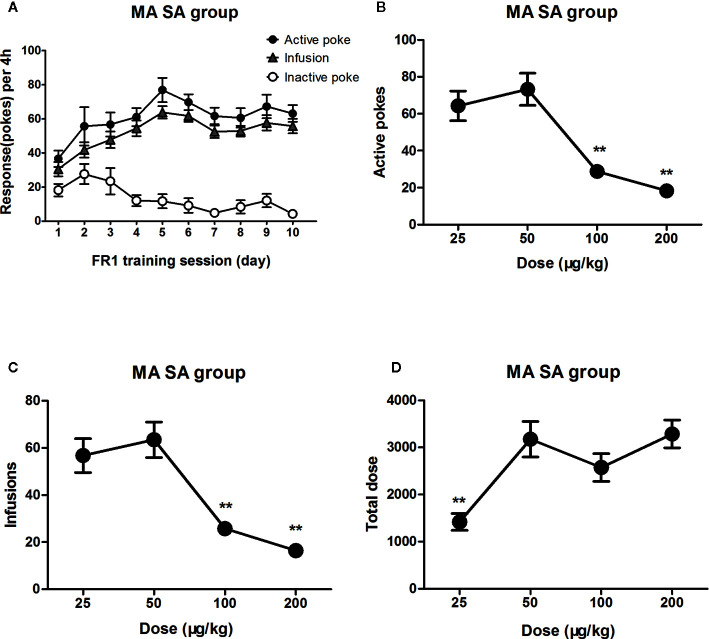
Dose–effect response in MA self-administration. Rats (n=16) were successfully trained to self-administer MA 4 h/day under an FR1 reinforcing schedule within 10 days **(A)**. When the MA trained rats were tested in different dose (25 μg/kg/infusion–200 μg/kg/infusion) of MA to self-administer 4 h in the following days (n=7-8/group). The active pokes and the infusions were changed and showed an inverted U trend. The number of active pokes and infusions decreased significantly at high MA doses (100 or 200 μg/kg/infusion) compared to 50 μg/kg/infusion doses **(B, C)**. Rat took less MA when dose decreased to 25 μg/kg/infusion, but maintain the total dose unchanged as dose increased (100, 200 μg/kg/infusion) **(D)**. **P < 0.01, compared to 50 μg/kg/infusion MA. Error bars = SEM.

Rats were successfully trained to self-administer MA under an FR1 reinforcing schedule as well (**Figure 3A**). When in dose–effect response in MA self-administration session, the statistical analyses indicated significant differences in terms of active pokes (F_3, 27_ = 17.5, *P*<0.01), infusions (F_3,27_ = 16.4, *P*<0.01), and total intake dose of MA (F_3,27_ = 8.5, *P*<0.01) at different training doses. *Post hoc* comparisons showed that active pokes and infusions decreased significantly at high MA doses (100 or 200 μg/kg/infusion) compared to 50 μg/kg/infusion doses (*P*<0.01). The dose–effect curve of MA also showed an inverted U-shaped trend distribution (**Figures 3B, C** **)**. However, only the total intake dose of MA decreased at low MA doses (i.e., 25 μg/kg/infusion) (*P*<0.01) (**Figure 3D**).

### The Dose–Effect Curve of Heroin Substitution for MA Reinforcement

**Figure 4:**
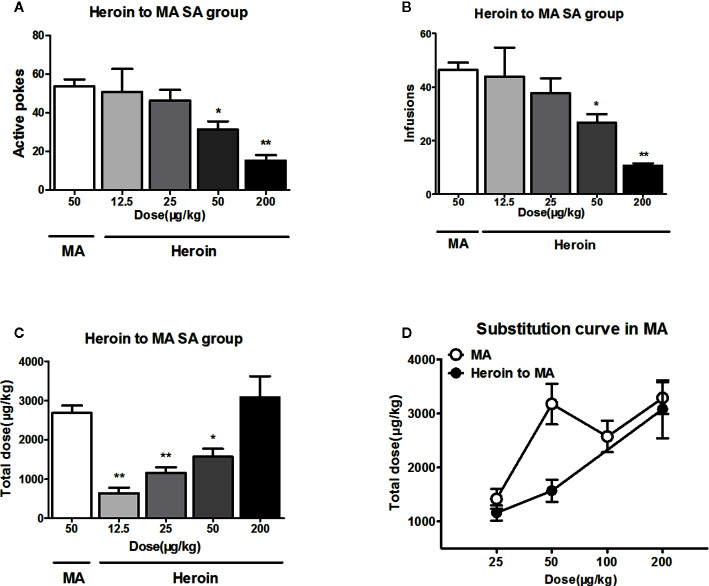
Heroin substituted for MA reinforcement. As substitution with different dose of heroin to self-administer 4 h in MA trained rats (n = 5–6/group), the higher doses (50, 200 μg/kg/infusion) of heroin significantly decreased the active pokes and the infusions **(A, B)**. The total intake dose of heroin significantly decreased at 50 μg/kg/infusion or below when replaced with heroin, and 200 μg/kg/infusion heroin could equally instead of 50 μg/kg/infusion MA self-administration at total dose **(C)**. The total dose–effect curve of heroin replacement showed shifted rightward and downward compared to the total dose–effect curve of MA **(D)** *P < 0.05, **P < 0.01, compared to 50 μg/kg/infusion MA. Error bars = SEM.

When the rats were allowed to self-administer different doses of heroin instead of 50 μg/kg/infusion of MA, the statistical analyses showed a significant change in the number of active pokes (F_4, 28_ = 5.8, *P*<0.01) and infusions (F_4, 28_ = 6.8, *P*<0.01). However, *post hoc* comparisons showed a significant decrease in the active pokes and infusions following heroin substitution at doses of 50 or 200 μg/kg/infusion (**Figures 4A, B** **)**.

Moreover, the total dose of heroin showed a progressive increase in MA trained rats. *Post hoc* comparisons indicated the total intake dose of heroin significantly decreased at 50 μg/kg/infusion or below when replaced with heroin. Only heroin doses of 200 μg/kg/infusion could equally substitute MA doses of 50 μg/kg/infusion (**Figure 4C**). Finally, compared to the total dose–effect curve of MA, the substituted curve shifted rightwards (**Figure 4D**).

## Discussion

According to these results, heroin and methamphetamine could replace each other during the self-administration training, suggesting that both opiates and psychostimulants may have a common rewarding mechanism. Besides, higher heroin doses seem to be necessary to replace the MA rewarding effects, whereas lower MA doses seem to be sufficient to replace the heroin rewarding effects.

With 4 h/d drug access, heroin or MA self-administration were stable both over time and across doses after 10-d training (15–18). The dose–effect curve of heroin showed a negative linear trend, and the active response decreased following the dose increase, consistently with the reinforcement theory (19).The limbic dopaminergic system was proposed as the neurobiological substrate for the rewarding effects of both opiates and psychostimulants. More specifically, heroin indirectly activates dopaminergic neurons in the ventral tegmental area (VTA), through inhibition of GABAergic interneurons (20), whereas MA directly redistributes dopamine from synaptic vesicles to the synaptic cleft, blocks dopamine reuptake, and inverts the direction of dopamine transport (8). According to the existing literature, both MA and heroin injection produce the release of dopamine in the NAc, as analyzed by microdialysis (21, 22). Further, more selective studies of drug-induced neuronal plasticity recently highlighted that these two drugs promote an overall reward circuitry signaling overlap, particularly through the modification of excitatory synapses in the NAc (23). Therefore, the common dopamine mechanism could explain the reciprocal substitutability between heroin and MA.

When heroin was replaced with MA, the total dose–effect curve shifted leftwards and upwards, possibly meaning that MA served as a heroin substitute; in addition, lower MA doses seem to be required to produce an identical or even more sensitive rewarding effect of heroin. Likewise, heroin could be replaced by a relatively lower dose of MA to reach are warding effect. On the other hand, when MA was replaced with heroin, the dose–effect curve shifted to the rightward, indicating that MA rewarding effect required a higher dose of heroin to be replaced.

Combining the substitution curves, the original heroin dose–effect curve was very similar to the dose–effect curve of MA substitution; likewise, the original MA and the curve of heroin substitution was also identical. The same trend in two separate experiments suggested that heroin and MA substitution was symmetrical, and their rewarding effect was independent. The phenomenon was supported by self-administration heroin and cocaine on alternate days, the escalated levels of cocaine self-administration fail to generalize heroin when change the heroin self-administrate back later (24). Similarly, the total dose of heroin or cocaine was not different from the total dose the rats intake which trained by single drug (heroin or cocaine) directly (25). The term “substitution” is typically used in drug discrimination procedures to determine if drugs share discriminative stimulus effects. For example, cocaine can partially substitute the discriminative stimulus effects of heroin in rats, and when heroin–cocaine combinations are administered to the heroin-trained rats, cocaine did not significantly alter the mean heroin dose–effect curve (26). Nonetheless, in another study with rhesus monkeys, opioids substituted the cocaine discriminative stimulus in some, but not in all of them (27). Some evidence showed that only small subsets of rats preferred either cocaine or heroin instead of the food alternative in the choice procedure, indicating the substance-specific pattern of drug preference (28). In several experiments, it was repeatedly shown that when the heroin trained rats resumed heroin training after being given MA instead of heroin, their heroin use was the same as before replacement with MA, indicating that the MA fully met the heroin demand during replacement days. Overall, these results suggest that MA could at least partly substitute heroin, and vice versa. Taken together, although psychostimulants and opioids seem to have their own independent way to substitute, MA and heroin did not affect the self-administration of the other.

Heroin and MA greatly differ in terms of pharmacological features as well as in clinical *manifestations* following their utilization (9, 10). The preclinical data indicated that the increase in the amount of dopamine induced by MA in the NAc is more than that induced by heroin. For example, when MA doses of 1 mg/kg were injected subcutaneously in rats, the levels of dopamine in their NAc increased up to 698% (29); however, when heroin doses of 0.2 mg/kg were injected intravenously in rats, the levels of dopamine in their NAc only increased up to 300% (30). Besides, gamma-vinyl GABA — an inhibitor of irreversible GABA-transaminase — only partially blocks the dopamine increase in the NAc caused by MA, yet completely inhibits the dopamine increase in the NAc caused by heroin (31). By contrast, heroin could enhance its rewarding effect through the CB1 receptor and other mechanisms independents of the dopamine levels in the NAc (21); further, MA also released large amounts of serotonin through its actions (22). Concerning cocaine, serotonin acts on the 5-HT_1b_ and 5-HT_2c_ receptors with bidirectional feedback to regulate the levels of dopamine in the NAc (32, 33). This indicates that the downstream reinforcement mechanisms of the two drugs are not the same. Therefore, MA and other psychostimulants may increase the levels of dopamine in NAc more significantly than heroin. That explained in part why low-dose MA could effectively replace heroin.

Previous clinical data showed a rather high frequency of combined utilization of MA and heroin in both habitual heroin and MA users (2, 5). Further, preclinical studies demonstrated that MA–heroin combinations (“speedballs”) produce greater rewarding effects (34); for example, urine of MMT patients in Yunnan tested positive for both morphine and MA (3), and half had used amphetamine among current primary heroin users while two-thirds of primary amphetamine users had used heroin (4). The main reasons for transition in drug use reside in either obtaining synergistic high or balancing out the effects of each other and keeping body functions as normal as possible (6). Another reason for drug transition is related to the price and availability of drugs: when MA prices increase, co-users choose to consume more heroin, and vice versa (35). The drug-market period in which injecting initiation occurred sensitively influenced the kind of first injected drug, thereby influencing some aspects of subsequent drug use in Australia (36).The present results provided the first direct evidence for the transition between MA and heroin use due to the reciprocal substitutability of their rewarding effects. The crisis of the global emergence of combining MA with heroin use is merging, and attention should be paid to their negative effects on public health (37). In conclusion, the present study demonstrated that heroin and MA can partly substitute each other; further, heroin and MA may follow their independent dose–effect mechanism during the substitution process.

## Data Availability Statement

The raw data supporting the conclusions of this article will be made available by the authors, without undue reservation.

## Ethics Statement

The animal study was reviewed and approved by Animal Ethics Committee of Ningbo University.

## Author Contributions

D-SM designed and performed research, analysis the data, and the writing of the paper. Y-JC, F-MW, and B-MM performed research and data collection. H-FL were responsible for guarantee of animal and experimental conditions. W-HZ and J-PX were responsible for the study concept and design, revising the paper, and study supervision.

## Funding

This work was supported by National Nature Science Foundation (81671321), National Key Research and Development Program of China Grant (2017YFC1310403), and Ningbo Natural Science Foundation (2019A610294) of China.

## Conflict of Interest

The authors declare that the research was conducted in the absence of any commercial or financial relationships that could be construed as a potential conflict of interest.
